# Validity evidence for the Hamburg multiple mini-interview

**DOI:** 10.1186/s12909-018-1208-0

**Published:** 2018-05-14

**Authors:** Mirjana Knorr, Anja Schwibbe, Maren Ehrhardt, Janina Lackamp, Stefan Zimmermann, Wolfgang Hampe

**Affiliations:** 1 Department of Biochemistry and Molecular Cell Biology, University Medical Center Hamburg-Eppendorf (UKE), N30, Martinistraße 52, 20246 Hamburg, Germany; 2 Department of General Practice / Primary Care, University Medical Center Hamburg-Eppendorf (UKE), W37, Martinistraße 52, 20246 Hamburg, Germany

**Keywords:** Multiple mini-interviews, Psychosocial competencies, Selection of medical students, Admission, Emotional intelligence, General practice

## Abstract

**Background:**

Multiple mini-interviews (MMI) become increasingly popular for the selection of medical students. In this work, we examine the validity evidence for the Hamburg MMI.

**Methods:**

We conducted three follow-up studies for the 2014 cohort of applicants to medical school over the course of two years. We calculated Spearman’s rank correlation (*ρ*) between MMI results and (1) emotional intelligence measured by the Trait Emotional Intelligence Questionnaire (TEIQue-SF) and the Situational Test of Emotion Management (STEM), (2) supervisors’ and practice team members’ evaluations of psychosocial competencies and suitability for the medical profession after a one-week 1:1 teaching in a general practice (GP) and (3) objective structured clinical examination (OSCE) scores.

**Results:**

There were no significant correlations between MMI results and the TEIQue-SF (*ρ* = .07, *p* > .05) or the STEM (*ρ* = .05, *p* > .05). MMI results could significantly predict GP evaluations of psychosocial competencies (*ρ* = .32, *p* < .05) and suitability for the medical profession (*ρ* = .42, *p* < .01) as well as OSCE scores (*ρ* = .23, *p* < .05). The MMI remained a significant predictor of these outcomes in a robust regression model including gender and age as control variables.

**Conclusions:**

Our findings suggest that MMIs can measure competencies that are relevant in a practical context. However, these competencies do not seem to be related to emotional intelligence as measured by self-report or situational judgement test.

**Electronic supplementary material:**

The online version of this article (10.1186/s12909-018-1208-0) contains supplementary material, which is available to authorized users.

## Background

Current competency frameworks in medical education agree that medical students and future doctors need pronounced psychosocial competencies such as communication skills, self-regulation and perspective taking [1–3]. Interest in considering these competencies already during the student selection process is growing [4]. Among the variety of selection methods used in medical education, multiple mini-interviews (MMI) are gaining popularity because of the superior reliability compared to traditional interview formats [5]. The MMI is an assessment method similar to an objective structured clinical examination (OSCE): candidates rotate through a circuit of several short stations and are assessed by one or two independent raters per station [5]. At each station, they usually respond to standardized questions or interact with a simulated patient. Unlike OSCEs, which primarily assess clinical skills, MMIs typically do not require the application of clinical skills or knowledge [5]. MMIs can be adjusted to specific objectives and therefore they differ among institutions in design and target competencies [6]. Three systematic reviews on MMIs have concluded that feasibility and internal reliability of MMIs are well researched and, amongst other points, that future research should focus more on validity evidence [6–8]. In this work, we describe the Hamburg MMI for undergraduate entry into medical school and summarise the first validity evidence for an MMI in a German context.

### Specification of the construct

#### Theoretical background

What MMIs measure is usually described by collective terms such as “non-cognitive variables” [5] and “pre-professionalism” [9]. Each MMI differs in the selection of domains that are subsumed under these general terms. Communication skills, ethical decision making, teamwork, integrity and empathy are examples of domains that are more frequent in the MMI literature [6]. While the term “non-cognitive” is still in use in the MMI literature, Roberts et al. [10] suggested early that MMIs do have a cognitive component.

As Eva et al. [11] pointed out, MMI scores should not be considered as mere representations of traits (i.e. dispositions of a person that are relatively stable over time) because the performance depends on the situation. This “context specificity” was the reasoning behind the multiple sampling approach of the MMI [5, 12]. The importance of the person-situation interaction is also emphasised in interactionist models of human behaviour such as the trait activation theory in the context of assessment centres [13, 14]. The key assumption of this theory is that situation trait relevance and situation strength determine whether and how traits are expressed in behaviour. It is expected that variability in trait-expressive behaviour is more likely in situations where trait relevant cues are present and behavioural demands and expectations are ambiguous [13]. In line with the general assumptions of interactionist theories [14], we suggest that verbal responses and observed behaviours at MMI stations are expressions of a complex structure of underlying traits and their interaction with the situation.

#### The Hamburg MMI

The MMI at the Hamburg medical school intends to assess psychosocial competencies and is used to select undergraduate medical students. We define “psychosocial competencies” in general as an effective regulation of the self and an effective interaction with others in critical interpersonal situations or when faced with typical problems that medical students and doctors will encounter throughout their career. Three focal domains guide the selection of station content: empathy, communication skills and self-regulation. We described the process that resulted in the selection of these three domains in an earlier publication [15]. The construct does not include clinical skills and it was further decided to exclude scenarios that require ethical reasoning.

We assume that a station score does not represent a candidate’s standing on a single domain but rather is a mixture of different domains. However, trait activation theory suggests that the selection of station content based on these three domains would lead to the presence of situational cues that would activate traits in the candidate related to these three domains. Altogether, the score of the Hamburg MMI is interpreted as a representation of a candidate’s psychosocial competencies in situations that predominantly require empathy, communication skills and self-regulation.

### Validity claims

#### Convergent validity evidence

All three of the MMI domains are multi-faceted constructs. A recent overview on empathy from a neuroscientific perspective, for example, proposes that empathy has four key components:

1. “Affective sharing, the first element of empathy to appear during ontogeny. It reflects the capacity to become affectively aroused by the valence and intensity of others’ emotions.
2. Empathic understanding, which entails the conscious awareness of the emotional state of another person.
3. Empathic concern, which refers to the motivation to care for someone’s welfare.
4. Cognitive empathy, similar to the construct of perspective taking or theory of mind is the ability to put oneself into the mind of another individual and imagine what that person is thinking or feeling” [16]

Following this definition, emotions and feelings of others play an important role in the empathy construct. Another construct that is closely related to aspects of empathy is emotional intelligence (EI). EI is described as “the ability to perceive and express emotion, assimilate emotion in thought, understand and reason with emotion, and regulate emotion in the self and others” [17], [p. 396]. Based on these definitions, both empathy and emotional intelligence encompass the understanding of emotions and the ability to perceive emotions in others.

If the MMI score is understood as a representation of psychosocial skills with empathy as one of the focal domains, we would expect a positive relationship with a measure of emotional intelligence. Although it plays a dominating role, empathy is not the only construct assessed by the MMI. Furthermore, empathy and emotional intelligence are not identical constructs despite the conceptual overlap. Thus, we assume that the relationship between MMI scores and the EI measure would be small in magnitude.

We know of only one study that analysed the relationship between MMI scores and an EI measure. In that study, the correlation between a self-report measure of EI and performance at an MMI for selection into allied health programs was non-significant [18]. Additionally, two other studies showed that EI was not related to traditional interviews [19, 20]. However, Libbrecht et al. found that EI correlated with the performance of medical students in practical courses on communication and interpersonal sensitivity which included role-plays with simulated patients [21].

#### Predictive validity evidence

The MMI is used for selecting students who will most likely demonstrate good psychosocial competencies in relevant situations throughout their medical career. In order to claim that the MMI fulfils this purpose, evidence is needed that MMI scores predict future behaviour in situations that evoke competencies related to empathy, communication skills and self-regulation.

The curriculum at the Hamburg medical school provides a first opportunity to test this claim after the first year of study. Before the start of the second year, students are required to spend one week with a general practitioner and assist with the daily routines. The general practice (GP) field provides a relevant context because in this field it is crucial that doctors possess good psychosocial competencies [22], perhaps even more than in other areas of the medical profession. General practitioners (GPs) work closely with patients and most patients will come regularly to the same doctor for consultation. Good psychosocial competencies help build trust and a positive doctor-patient relationship. This positive relationship is highly relevant to GPs and patients and leads to positive outcomes [23–25]. For this reason, the second study analyses the GPs’ evaluations of the students’ psychosocial competencies. We expected a positive correlation between MMI scores and GPs’ evaluations. Unlike the standardised setting of the MMI, the experience and situations during the week in the practice can vary from student to student and evoke psychosocial competencies to a different degree. Therefore, we assumed that the relationship would be small to medium in size.

Three studies have reported that performance in an MMI or MMI-like interview procedure could predict evaluations by supervisors or peers during clerkship [26–28]. Similarly, MMIs for the selection into specialty training could predict supervisor ratings of communication skills [29]. However, Burkhardt et al. found that the MMI was not significantly correlated with first-year resident performance when other selection factors were included in the model [30].

The second possibility for analysing the predictive evidence in Hamburg is a standardised OSCE after one and a half years of study. While the OSCE is primarily designed to assess clinical skills, it includes stations in which communication skills are assessed to a varying degree. The MMI and the OSCE in Hamburg are based on the same understanding of good communication skills [31]. We assumed that there would be a small positive correlation between the MMI and OSCE because both intend to measure communication skills to a differing extent. We further expected that there should be a stronger relationship to OSCE stations with a focus on these skills.

The MMI literature so far has reported correlations with OSCE performance or other performance-based exams of −.05 < *r* < .43 [26, 32–35].

#### Control variables

For each of the three studies, we considered age, gender and other selection measures as possible control variables. Both gender and age could be argued to have an influence on psychosocial competencies. There are indications that women perform better on measures of empathy and communication skills; for example, in the context of an OSCE [36]. Research in neuroscience suggests that these differences might have evolutionary explanations [37]. Although the results of studies on gender differences in MMI scores are mixed [8], Ross et al. recently demonstrated an advantage for female applicants in a cumulative meta-analysis at the University of Calgary [38]. In addition, maturity could play a role in the development of psychosocial competencies. Older candidates might have gained more life experience that could help them develop more confidence in psychosocial competencies. Again, the results on the relationship between age and MMI performance are mixed but if a significant relationship is found, it is positive and rather small [8]. Finally, the MMI can only be considered as a useful selection tool if it predicts relevant outcomes over and above the existing selection measures.

### Relevance

Although all three criteria (EI, supervisor ratings, OSCE scores) have already been the subject of previous studies, this is the first study to report validity evidence for a German MMI. Furthermore, the relationship with emotional intelligence has only been analysed in one previous study, which focused on an MMI for selection into allied health sciences programs. This work also provides further insight by concentrating on the GP context as a field in which psychosocial competencies are considered highly relevant. Overall, the accumulated validity evidence for different MMIs suggests that MMIs can be designed to be predictive of relevant outcomes. However, the results are sometimes mixed and they depend on the design of the MMI. More insight is needed regarding the theoretical framework of MMIs and their design that would help to explain why results are sometimes positive and sometimes negative [6]. Therefore, this work provides details regarding the underlying construct and the design of the MMI. We aim to provide information that could be relevant in future comparisons of different MMI approaches and their validity evidence.

## Methods

### Cohort and selection procedure

In this study, we analysed data from the 2014 admission cohort at the University Medical Center Hamburg-Eppendorf. Forty percent of places at medical schools in Germany are assigned by a centralised agency and based on students’ high-school grade point averages (GPA; German: Abitur), waiting time and other criteria. For the other 60% of places, each university has its own local admission procedure. In Hamburg, candidates were pre-selected based on their GPA and had to write a natural science test (HAM-Nat) [39]. The first 115 candidates in a ranking order based on their GPA and HAM-Nat results were directly rewarded with a study place. The next 200 applicants in the ranking were invited to an MMI [15]. The selection decision was based on the following procedure: GPA values were transformed into a linear 60-point scale, while HAM-Nat and MMI were each transformed into a linear 59-point scale. The sum of these three scores determined the ranking order for the remaining 106 study places. Given that MMI participants were pre-selected by a combination of GPA and HAM-Nat, the range of these two combined factors was small (range: 79–87 points), which allowed the MMI scores (range: 24–50 points) to have a stronger impact on the final ranking.

### Multiple mini-interview

The 2014 interviews took place on a single day with four parallel circuits in four consecutive rounds. Candidates rotated through seven stations including three stations with an interviewer and four stations with simulations. The overall performance at each station was evaluated by two raters on a Likert-scale ranging from 1 (very poor) to 5 (very good). The mean over these two ratings was taken to form the station score. A candidate’s overall MMI score was then calculated as the mean of all station scores. Raters were medical doctors, psychologists and other employees of the university’s medical centre. A 4-h rater training took place one day before the interviews and consisted of a general introduction to the interview procedure as well as station-specific training, which included mock interviews. Raters were provided with detailed descriptions about the station task and a score sheet that listed 9 to 14 behavioural descriptors of a very poor and a very good performance that were structured under three to five station-specific rating categories. Table 1 gives an overview of station tasks including examples of behavioural descriptors related to empathy and communication skills.

**Table 1:** Overview of MMI stations

Station number	Type	Main task	Example of behavioural descriptors on score sheets related to empathy and communication skills
1	Simulation	find a balance between one’s own interests and another person’s needs and motives	• recognises SP’s loneliness and the need for company • active listening
2	Interview	respond to a past-behavioural question concerning a situation in which the candidate felt frustrated	• thinks about possible reasons, motives and feelings of the other persons involved; coherently justifies his/her thoughts • structures his/her description
3	Simulation	reject a request of a worried patient	• demonstrates sympathy for the SP’s worries about his/her partner and for the insecurity of not knowing the diagnosis • communicates clearly
4	Interview	describe observations made in a video of an interaction between a mother and a son	• considers the structure of the relationship between the two in his/her analysis • descriptions are precise, […] to the point
5	Simulation	address a conflict (in a monologue)	• demonstrates appreciation for the other person (e.g. empathises positive sides and similarities, expresses wishes, takes the other person’s perspective) • describes clear motives
6	Simulation	shared-decision making	• expresses sympathy for feelings (e.g. desire for independence, disappointment / anger because of family, fear) • speaks clearly, slowly and comprehensibly
7	Interview	reflect on and interpret emotional statements by patients	• names emotions and thoughts • description is vivid and allows for a good understanding of the emotional situation

*SP* = simulated patient

### Study measures

#### Measures of emotional intelligence (EI)

Three months after the admission procedure we asked all admitted and rejected candidates of the local selection process to participate in an online study that included two EI measures. As there is an ongoing dispute in the literature regarding the conceptualisation and measurement of EI [40, 41], each of the two measures represented one of the two dominant streams within EI research. One measure conceptualises EI as a trait (trait EI) and the other conceptualises EI as an ability (ability EI). As the trait EI measure relies on self-report items, it is particularly prone to “faking good” tendencies that have been observed in high-stake selection procedures [42, 43]. Therefore, we chose a three-month time lag to ensure that candidates would not associate the questionnaire with the selection process. Participants could win one of ten 50 EUR vouchers for an online bookstore.

*Trait Emotional Intelligence Questionnaire Short Form (TEIQue-SF).* The TEIQue-SF [44, 45] is a 30-item self-report questionnaire that measures global trait EI. An example item is “I can deal effectively with people”. Participants rate their level of agreement for each item on a scale of 1 (completely disagree) to 7 (completely agree). A validated German translation was available from the test developers [46, 47]. Previous studies reported internal consistencies of α > .80 [44, 47]. A recent systematic review summarised that the TEIQue-SF explains incremental variance in criteria such as academic performance and life satisfaction (.01 ≤ ΔR^2^ ≤ .18) over and above higher-order personality dimensions such as the Big Five [48]. In their systematic review for the medical education field, Arora et al. [49] recommended the use of the TEIQue in further EI research because of the good psychometric properties of the test.

*Situational Test of Emotion Management (STEM).* The STEM [50] measures emotion management, which is one facet of ability EI. It is a situational judgement test (SJT) with 44 scenarios. Each scenario describes an emotional situation (e.g. “Surbhi starts a new job where he doesn’t know anyone and finds that no one is particularly friendly.”) and includes four possible responses (e.g. “Have fun with his friends outside of work hours.” / “Concentrate on doing his work well at the new job.”). Test takers are asked to either select the most effective response (multiple-choice) or to rate the effectiveness of each response (rate-the-effectiveness) and their ratings are compared to ratings by subject matter experts [50]. A validated German translation of the STEM was available by Hilger et al. [51]. According to this version, we asked candidates to rate the effectiveness of each response on a scale ranging from 1 (not effective at all) to 6 (very effective). The candidate’s final scores were calculated as the mean of the absolute difference from an expert rating for each item. Therefore, small values of the STEM indicate stronger agreement between the participant and the subject matter experts.

Internal consistencies for both formats were reported to be acceptable (α = .68 for multiple-choice, α = .92 for rate-the-effectiveness) [50]. Previous studies have demonstrated that the STEM scores do not reflect personality or fluid intelligence [50, 52, 53]. It is related to other performance-based measures of EI but shows low to non-significant correlations to self-reported EI [53, 54]. Evidence for the validity of the assumptions of the STEM was further demonstrated by negative correlations with externally oriented thinking and states of distress as well as positive correlations with retrospective life satisfaction, academic achievement and well-being [50, 55]. Most importantly, the STEM could predict performance in courses on communication and interpersonal sensitivity during the first three years of medical school [21].

#### General practitioners’ evaluations

After the first year of study, all students spend one week with a general practitioner (GP). The major goal of this 1:1 teaching is to introduce at an early stage the students to the GP field. Therefore, students mainly watch and assist when possible. They are also given the opportunity to take patients’ histories and perform physical examinations. A few weeks prior, we asked students for their consent to collect data and match it to admission data. Participating students could win one of five 100 EUR vouchers for an online store.

All general practitioners involved in the study were at the time part of a GP teaching program at the university medical centre. Practices can take one or more students but only one student at a time. Some of the GPs took part in an information session. All GPs received a written explanation of the study and instructions on how to complete the questionnaire. For each student, GPs received a letter at the end of the week with three questionnaires. We asked GPs and up to two other members of their practice team who interacted with the student to complete the questionnaire.

A literature research did not identify any existing instrument that was suitable for our study purposes especially considering our aim – which was to focus on psychosocial competencies – and also the limited amount of time that GPs had to complete the questionnaire. Therefore, two of the authors (MK and AS), both psychologists, developed a new questionnaire that was reviewed by a third author (ME) – who is a general practitioner and coordinates the teaching practices program – and a medical student. Two validated instruments in German for the evaluation of encounters with patients provided ideas for relevant rating categories: the Frankfurt Observer Communication Checklist (FrOCK) [56] and the Globalskalen ÄGF-A [57]. As we needed an instrument for evaluating a whole week and with a focus on psychosocial competencies, we left out rating categories that were applicable to only single encounters (e.g. the structure of the conversation) and categories that concentrated on clinical aspects (e.g. history taking). In addition, a student assistant of the admission research group collected accounts of experiences of fellow second year medical students after they had finished their general practice week. Based on these accounts, the student assistant provided a list of possible observable behaviours in encounters with patients and members of the practice. We then compared the categories from the literature with the accounts by the students and created five categories under which we structured the behavioural descriptors. The categories were “conversation”, “non-verbal communication”, “interaction”, “work habits” and “professionalism and teamwork” (Additional file 1). Each category was rated on a five-point Likert scale ranging from 1 (very weakly pronounced) to 5 (very well pronounced). In an attempt to draw a connection between the selection context of the MMI and the GP context, we additionally asked the raters to give an evaluation of the suitability of the student for the medical profession on a four-point Likert scale ranging from 1 (“not suitable”) to 4 (“absolutely suitable”).

### OSCE

Students can register for the first OSCE after one and a half years of study, at the earliest. It consists of twelve stations, which mostly assess basic clinical skills such as measuring blood pressure, interpreting radiographic images, and examining lungs. One of the stations primarily measures communication skills. Learning objectives that are tested at this station include structuring a conversation meaningfully, verbalizing emotions adequately, and paraphrasing the patient’s subjective concept of the illness. In addition, two other stations on history taking indirectly rely on communication skills. Common learning objectives that are tested at both of these stations are patient-centred communication and the structure of history taking. Students can receive up to 20 points for each station resulting in a maximum possible overall OSCE score of 240 points. We analysed the correlation between overall MMI score and overall OSCE score and, more specifically, between MMI score and the performance at each of the three communication and history-taking stations.

### Statistical analysis

The statistical analysis was conducted in IBM SPSS Version 21.0.0.0 and R version 3.4.2 including the packages RVAideMemoire and MASS. For the analysis of our hypotheses, we first calculated all zero-order correlations between MMI performance and the outcome variables. If a significant relationship was found, its stability was further investigated in a regression model for each of the outcomes. To determine which of the possible control variables had to be considered in the model, we analysed whether gender, age, HAM-Nat result and GPA were related to MMI performance or any of the outcomes. The final regression models would then include MMI performance and all relevant control variables as predictors.

### Ethics

The local ethics commission board (Ethik-Kommission der Ärztekammer Hamburg, PV4983) approved this research as not constituting research with human subjects (“kein Forschungsvorhaben am Menschen”) in a clinical sense. All students who were included in the analysis gave their written informed consent to collect, match and analyse their admission and study data.

## Results

### Participants

Table 2 lists the number of candidates in 2014 and the participation rates for each study. Twenty-four percent of the candidates who were invited to the EI online study participated and provided a full dataset, including 35% of candidates who underwent the MMI. These were 38 out of 106 candidates who were admitted by the MMI and 31 out of 90 candidates who were rejected after the MMI.

**Table 2:** Number of candidates/study participants, mean age at each time point and gender for total 2014 cohort and respective study, including time of data collection

	Total cohort (09/2014)	Study 1 (12/2014)	Study 2 (10/2015)	Study 3 (09/2016)
	Admission process	EI online	GP evaluations	OSCE
Admitted				
Central agency quota (*N*)	164	―	76	115
HAM-Nat quota (*N*)	115	41	60	97
MMI quota (*N*)	106	38	59	89
Age (*M* / *SD*)	22.6 / 4.9	20.8 / 2.1	23.0 / 3.9	23.4 / 3.5
Gender (percentage male)	39.7%	43.0%	36.4%	40.9%
Rejected				
HAM-Nat quota (*N*)	720	137	―	―
MMI quota (*N*)	90	31	―	―
Age (*M* / *SD*)	20.3 / 1.8	20.6 / 2.2	―	―
Gender (percentage male)	32.2%	28.6%	―	―

*N* = number of students*, M* = mean, *SD* = standard deviation

EI = emotional intelligence, GP = general practice, OSCE = objective structured clinical examination, HAM-Nat = Hamburg natural science test, MMI = multiple mini-interview

At the time of the GP study, 281 out of 385 students (73%) were registered for a general practice and agreed to participate. The response rate by the practices was 77% (217 out of 281). Finally, GP study data from 195 participants could be matched to the 2014 admission data including 59 students who undertook the MMI (56% of all who were admitted after the MMI). In two cases, raters did not provide a suitability rating.

OSCE data was available for 301 students who had already gained the required study achievements and who had registered for the exam in 2016. Out of these, 89 had MMI performance data (84% of all who were admitted after the MMI).

### Descriptive statistics

We observed high means and negative skewness for all variables except for the STEM, which is the only variable where lower values signify better performance (Table 3). In addition, Shapiro-Wilk tests suggested that normality cannot be assumed for any of the variables. The descriptive statistics indicated that small sample sizes, deviations from the normal distribution and the presence of outliers required statistical methods that were robust against these conditions. Therefore, Spearman’s rank correlation was used to analyse the relationship between two variables and Wilcoxon rank-sum tests to analyse group differences.

**Table 3:** Descriptive statistics and reliability values for study measures

	*N*	*M*	*SD*	*Min / Max*	*Skewness*	*Shapiro-Wilk Test*	*Reliability*
MMI	196	3.63	.47	2.38 / 4.69	−.35	W = 0.99, *p* < .05	Overall = .65^a^: *ICC* = .68
Study 1:							
Global Trait Emotional Intelligence (TEIQue-SF)	247	5.38	.62	3.57 / 6.63	−.51	W = 0.98, *p* < .001	*α* = .86
Emotion Management (STEM)	247	1.11	.23	.71 / 2.38	1.59	W = 0.90, *p* < .001	*α* = .94
Study 2:							
GP psychosocial competencies	195	4.53	.72	1.00 / 5.00	−2.46	W = 0.68, p < .01	*α* = .94^b^: *ICC* = .88 / .92^b^
GP suitability	193	3.71	.54	1.00 / 4.00	−2.20	W = 0.60, *p* < .01	*ICC* = .73 / .61^c^
Study 3:							
OSCE	301	199.65	10.17	164 / 221	−1.13	W = 0.93, p < .001	*α* = .53^d^

*N* = number of students*, M* = mean, *SD* = standard deviation, *Min / Max* = minimum and maximum value, *α* = Cronbach’s Alpha, *ICC* = intraclass correlation for interrater reliability

MMI = multiple mini-interview, TEIQue-SF = Trait Emotional Intelligence Questionnaire Short Form, STEM = Situational Test of Emotion Management, GP = general practitioner, OSCE = objective structured clinical examination

^a^Overall reliability was estimated by means of a mixed-model. See Hissbach, et al. [15] for more details on the formula

^b^Alpha refers to the internal consistency of five items. ICCs refer to the interrater-agreement for the average measure of the five items in cases of two raters (first value, *n* = 53) and in cases of three raters per subject (second value, *n* = 74)

^c^Interrater-agreement for one item in cases of two raters (first value, *n* = 51) and in cases of three raters per subject (second value, *n* = 74)

^c^Alpha refers to the internal consistency of twelve station scores

The reliability of each measure was determined using Cronbach’s alpha for internal consistencies, intraclass correlations for interrater reliability and a mixed model approach for the overall generalisability of the MMI result. A detailed description of this approach can be found in an earlier publication [15]. The overall reliability of the MMI was .65 and the overall interrater agreement was ICC = .68. Both values are within the range of what is typically reported in the MMI literature [6]. Cronbach’s alpha values for both of the EI measures were > .80. The internal consistency for the five social competency items in the GP study was very high (α = .94). Therefore, we used the mean of these five items over one to three raters (“GP psychosocial competencies rating”) as the outcome measure for the study. The second outcome measure was an evaluation of the suitability for the medical profession (“GP suitability rating”) over one to three raters. Both outcome measures showed enough variability to allow for a cautious comparison to admission data. The raters of the GP study were medical doctors (63.8%), medical assistants (30.9%) and practice team members of other professions (1.3%). Four percent of the raters did not make any statement concerning their profession. Wilcoxon rank-sum tests showed that ratings by practices of physicians who were present at the information session (25% of participating practices) did not differ significantly from ratings by practices that did not attend (GP psychosocial skills: W = 52,608, *p* > .05; GP suitability: W = 24,720.5, *p* > .05). Finally, the internal consistency of the OSCE stations was α = .53.

### Control variables

There was a small but significant correlation between age and MMI result (ρ = .16, *p* < .05). Age was also significantly related to GP psychosocial skills (ρ = .17, *p* < .05) as well as GP suitability (ρ = .23, *p* < .01). A Wilcoxon rank-sum test revealed a significant difference in MMI scores for gender (W = 5781, *p* < 0.01) with female candidates scoring higher (Md = 3.74) than male candidates (Md = 3.55). No significant gender differences were found for any of the outcome variables. Neither GPA nor HAM-Nat performance was related to MMI or any of the outcome variables (Additional file 2).

### Correlations between MMI performance and outcome measures

Neither global trait EI nor emotion management (ability EI) correlated with MMI results (Table 4). However, MMI results correlated significantly with GP psychosocial competencies (ρ = .32, *p* < .05), GP suitability (ρ = .42, *p* < .01) and OSCE score (ρ = .23, *p* < .05). While the confidence interval for both the correlation with GP psychosocial competencies as well as OSCE score had a lower bound close to zero, this value was higher for the correlation with GP suitability (CI_lowerbound_ = .17). There were no significant correlations between MMI score and specific scores on the three OSCE stations, which directly or indirectly focus on communication skills (communication: ρ = .14, *p* > .05, 95% CI: −.07/.34; history taking 1: ρ = .02, *p* > .05, 95% CI: −.20/.22; history taking 2: ρ = .13, *p* > .05, 95% CI: −.08/.34).

**Table 4:** Spearman’s rank correlation between MMI and study measures

	1. MMI	2. Trait EI (TEIQue-SF)	3. Emotion Management (STEM)	4. GP psychosocial competencies	5. GP suitability
**Study 1:**					
2. Trait EI (TEIQue-SF)	.07 (−.19/.30), *n* = 69				
3. Emotion Management (STEM)	.05 (−.17/.27), *n* = 69	.16* (.03/.28), *n* = 247			
**Study 2:**					
4. GP psychosocial competencies	.32* (.04/.55), *n* = 59	−.14 (−.43/.15), *n* = 44	−.07 (−.38/.25), *n* = 44		
5. GP suitability	.42** (.17/.64), *n* = 58	−.15 (−.42/.15), *n* = 44	.01 (−.31/.36), *n* = 44	.71** (.62/.79), *n* = 193	
**Study 3:**					
6. OSCE	.23* (.03/.41), *n* = 89	.12 (−.13/.34), *n* = 67	−.06 (−.30/.19), *n* = 67	.08 (−.08/.24), *n* = 181	.15 (−.002/.29), *n* = 180

* *p* < .05, ** *p* < .01

95% confidence interval in parentheses

*MMI* = multiple mini-interview, *EI* = emotional intelligence, *TEIQue-SF* = Trait Emotional Intelligence Questionnaire Short Form, *STEM* = Situational Test of Emotion Management, *GP* = general practice, *OSCE* = objective structured clinical examination

### Robustness of the results

In a final step, we analysed whether the positive correlation between MMI and GP psychosocial skills, GP suitability and OSCE overall score would be sustained when controlling for the identified relevant control variables of gender and age. For this analysis, we used robust regression models with an M estimator. Robust regression is advisable if the assumptions of linear regressions are violated, particularly in the case of outliers [58]. The robust regression models for each of the three outcomes showed that the MMI remained a significant predictor when the control variables were part of the model. Both gender and age were non-significant predictors in each of the models (Table 5).

**Table 5:** Robust regression analyses including control variables

Dependent variable	Independent variable	*b*	*SE*	*t*	*df*
GP psychosocial skills	MMI	0.53	0.20	2.67*	54
	gender	−0.12	0.11	−1.06	54
	age	0.01	0.04	0.15	54
GP suitability	MMI	0.65	0.17	3.77**	53
	gender	−0.11	0.10	−1.11	53
	age	0.001	0.03	0.06	53
OSCE overall score	MMI	7.05	3.46	2.04*	85
	gender	−2.29	1.98	−1.15	85
	age	0.005	0.58	0.01	85

* *p* < .05, ** *p* < .01

*b* = robust regression coefficient, *SE* = standard error, *df* = degrees of freedom

*MMI* = multiple mini-interview, *GP* = general practitioner, *OSCE* = objective structured clinical examination

## Discussion

The growing implementation of resource-intensive MMIs into the admission procedures of medical schools around the globe demonstrates that the psychosocial competencies of future medical students are highly valued. Evidence that MMIs really measure these competencies is still needed [4, 6]. We hypothesised that if the MMI scores could be interpreted as measures of psychosocial competencies with empathy as one of the focal domains, there would be a positive relationship between MMI performance and EI. However, we did not find a significant correlation with either of the EI measures.

We replicated the finding by Yen et al. [24] that MMI performance does not correlate with a self-report measure of EI. Although we administered the questionnaire three months after the admission process, it might be that candidates still felt the need to present themselves favourably. It is known that self-ratings generally tend to be inflated and influenced by leniency and social desirability biases [59]. Moreover, “faking good” in personality measures is a well-observed phenomenon in the context of medical school selection [42, 43]. If self-perceptions of emotion related competencies did not match observer perceptions, this could explain the missing relationship.

However, we also could not find a positive correlation with emotion management. The STEM is a performance-based measure of EI in which a person’s response is compared to responses by experts. This measure thereby puts an emphasis on the perspective of others on emotional intelligent behaviour, similar to raters in an MMI. It could be that emotion management in particular is too narrow a construct for the broad construct of psychosocial competencies that we want to measure in our MMI. If we look for commonalities between the definitions of empathy and emotional intelligence, both rely on understanding emotions and the ability to perceive emotions in others. One could argue that perceiving and understanding emotions are necessary for effectively managing emotions. However, we suggest further exploration of this matter and advise that future studies consider alternative ability EI measures such as the Mayer-Salovey-Caruso Emotional Intelligence Test (MSCEIT) [60], which includes subtests related to the perception and understanding of emotions. Finally, our finding could also be due to a methodological artefact of the response format. Many of the published validity studies on the STEM relied on the multiple-choice format [21, 53–55], although the rate-the-extent format is superior in reliability [50]. We decided to use the rate-the-extent format of the German version because it provides more information than the multiple-choice format. There is no clear explanation yet for the difference between the response formats [50]. If further studies on the relationship between MMI performance and the STEM are conducted, it is advisable to take both response formats into account.

In line with our expectation, MMI results correlated with ratings of psychosocial competencies in a GP context. This finding supports the intended use of the MMI to select medical students who will demonstrate psychosocial competencies in relevant contexts. However, the finding is preliminary and there are some weaknesses in this study. We did not have the opportunity to provide all of the raters with intensive training as a means to make sure that the frame of reference was the same for every rater. This could explain why ratings were in general very favourable. Both MMI scores and GPs’ evaluations were based on observational ratings. Although we provided instruction and objective rating criteria in both cases, subjective impressions surely played an additional role in both rating processes. The GP suitability ratings were even conceptualised as an overall subjective impression. Looking at the confidence intervals, the relationship between MMI scores and GP suitability ratings seems more trustworthy than the relationship between MMI scores and GP ratings of psychosocial skills. This could mean that the positive correlation between MMI scores and GP ratings are due rather to similar subjective impressions than similar objective rating criteria. A further limitation of this study is the use of a newly developed questionnaire rather than a validated measure. We could not find a previously validated tool that would fit our purpose of concentrating on psychosocial competencies only. We worked closely with GPs and medical students who had already spent a week in a general practice to make sure that the questionnaire was tailored to the conditions and possibilities of the GP week.

This study replicates findings that MMI performance predicts OSCE performance. Yet our expectation that this relationship should be stronger for stations that focus on communication skills was not supported. It is possible that the MMI assesses a more subjective impression of general good communication skills while the OSCE communication station more strongly focuses on the objective evaluation of the correct application of communication techniques in a physician-patient interaction that are taught in medical school. We assumed that OSCE history taking stations indirectly assess communication skills but the indirect effect on these stations’ scores might have been too small. Overall, the OSCE puts an emphasis on clinical skills that were not assessed in the MMI. Therefore, the correlation between MMI and OSCE could be due to similar, more general competencies that are required to handle an exam situation well [33]. However, the lower bound of the correlation between MMI and OSCE score was close to zero which indicates that this correlation has to be interpreted with caution.

There are three general limitations to this study. First, we had drop-out rates for all three studies. Only MMI participants who were admitted could participate in the GP and OSCE studies. The response rates for the MMI subgroup in these studies were acceptable (GP study: 56%, OSCE study: 84%). However, while we were able to invite admitted and rejected MMI participants to the EI online study, only 35% of them responded to the questionnaire. Given that the questionnaire was administered online the lower response rate is not surprising and is comparable to similar studies [61, 62]. Second, results are impaired by range restriction [63] because candidates with low MMI values were not admitted. However, this limitation most likely weakens the correlations between MMI performance and GP ratings or OSCE scores. It is therefore probable that the actual correlations are even higher. Moreover, range restriction could also be one reason for the non-association between MMI results and OSCE communication stations. The third general limitation is that we only analysed one cohort at one medical school over the course of two years. Further follow-up studies are under way in which we will analyse whether our findings are robust over cohorts and for later time points. Multi-centre studies based on the same MMI would allow to further test the generalisability of evidence regarding the validity of the MMI.

## Conclusion

In this study, we inspected three criteria that we expected to be related to MMI performance. The strongest association was found in a practical context with the evaluation of students’ suitability for the medical profession. Here, the medical students worked in a multi-professional team and formed initial relationships with real patients without the pressure of an exam. The Hamburg MMI was not developed to primarily predict good performance at exams or personality tests, but rather to predict behaviour in a real-life professional context. Therefore, this finding provides preliminary support for the assumption that we can generalize from the behaviour in the MMI context to a professional context where psychosocial competencies are very relevant. Future validation studies need to investigate further associations with desired outcome criteria, particularly with regard to patients’ perceptions of the medical student. While we could find predictive validity evidence for the Hamburg MMI, it remains uncertain whether the claim that MMI scores represent psychosocial competencies as defined in the introduction holds true. The results indicate that more elaboration on a comprehensive theory for psychosocial competencies and the MMI as an assessment tool is necessary.

## Additional files

Additional file 1: Appendix 1, Research questionnaire, English translation of the questionnaire which was developed for the general practice study (DOCX 30 kb)

Additional file 2: Appendix 2, Tests for control variables, Statistical analyses of the relationship between the control variables (gender, age, GPA and HAM-Nat) and study variables (MMI and outcome measures) (DOCX 14 kb)

## Abbreviations

EI: Emotional intelligence
GP: General practice
GPA: Grade point average
HAM-Nat: Hamburg natural science test
MMI: Multiple mini-interview
OSCE: Objective structured clinical examination
STEM: Situational Test of Emotion Management
TEIQue-SF: Trait Emotional Intelligence Questionnaire
