# Supplementary material for: Validity evidence for the Hamburg multiple mini-interview
Source: BMC Med Educ. 2018 May 14;18:106. doi: 10.1186/s12909-018-1208-0 (PMC5950198; doi:10.1186/s12909-018-1208-0)
Supplement: Supplementary file 1 — Appendix 1, Research questionnaire, English translation of the questionnaire which was developed for the general practice study (DOCX 30 kb) [file 12909_2018_1208_MOESM1_ESM.docx]

**Research questionnaire**

The following English questionnaire is a rough translation of the original German questionnaire. Words and expressions should be revised critically by the researcher before used in practice.

Please do not show this questionnaire to the student! The admission research group will not inform students, the deanery or the Institute of Primary Care about individual ratings.

**Name of the student:**

**Your profession:** physician  medical assistant  other ____________________

| **Conversation**   - listens carefully - expresses himself/herself clearly and comprehensibly - demonstrates interest and openness - asks questions |  |  |  |  |  |  |
| --- | --- | --- | --- | --- | --- | --- |
| **Non-verbal communication**   - holds good eye contact - has appropriate posture - adequately balances closeness and distance |  |  |  |  |  |  |
| **Interaction**   - behaves politely and respectfully - is pleasant, creates a pleasant atmosphere |  |  |  |  |  |  |
| **Work habits**   - makes use of opportunities to assist - understands instructions and is able to put them into practice |  |  |  |  |  |  |
| **Professionalism and teamwork**   - demonstrates appropriate behavior towards patients and the practice team - behaves according to his/her role - observes rules (e.g. dress code, punctuality, etc.) - assimilates well to the daily routines of the practice |  |  |  |  |  |  |

You can base the evaluation of the listed competencies on all observations made over the week. Situations can include interactions with you, practice team members or patients.

**Not applicable**

**very weakly pronounced**

**very well pronounced**

**averagely pronounced**

Please imagine this would be a selection procedure. Based on your personal impression, **how suitable is the student for the medical profession in your opinion?**

| **absolutely suitable** | **rather suitable** | **less suitable** | **not suitable** |
| --- | --- | --- | --- |
|  |  |  |  |

**Thank you for your evaluation!**

You can use the back of the questionnaire for open comments and remarks.
