# Supplementary material for: Validity evidence for the Hamburg multiple mini-interview
Source: BMC Med Educ. 2018 May 14;18:106. doi: 10.1186/s12909-018-1208-0 (PMC5950198; doi:10.1186/s12909-018-1208-0)
Supplement: Supplementary file 2 — Appendix 2, Tests for control variables, Statistical analyses of the relationship between the control variables (gender, age, GPA and HAM-Nat) and study variables (MMI and outcome measures) (DOCX 14 kb) [file 12909_2018_1208_MOESM2_ESM.docx]

**Tests for control variables**

**Gender**

| Gender differences for | Wilcoxon rank-sum test |
| --- | --- |
| MMI | W = 5781, p < 0.01 |
| Trait EI (TEIQue-SF) | W = 6210.5, p > .05 |
| Emotion management (STEM) | W = 6562, p > .05 |
| GP psychosocial skills | W = 4256, p > .05 |
| GP suitability | W = 4481.5, p > .05 |
| OSCE | W = 10504, p > .05 |

**Age**

| Correlation between age and… | Spearman’s rho | 95% Confidence Interval | n |
| --- | --- | --- | --- |
| MMI | ρ = .16* | .02/.30 | 196 |
| Trait EI (TEIQue-SF) | ρ = -.09 | -.21/.03 | 247 |
| Emotion management (STEM) | ρ = -.04 | -.16/.09 | 247 |
| GP psychosocial skills | ρ = .17* | .01/.30 | 195 |
| GP suitability | ρ = .23** | .09/.35 | 193 |
| OSCE | ρ = -.06 | -.17/.06 | 301 |

**GPA**

| Correlation between GPA and… | Spearman’s rho | 95% Confidence Interval | n |
| --- | --- | --- | --- |
| MMI | ρ = -.13 | -.26/.01 | 196 |
| Trait EI (TEIQue-SF) | ρ = -.11 | -.23/.01 | 247 |
| Emotion management (STEM) | ρ = -.01 | -.14/.11 | 247 |
| GP psychosocial skills | ρ = .08 | -.05/.22 | 195 |
| GP suitability | ρ = .13 | .00001/.24 | 193 |
| OSCE | ρ = -.10 | -.21/.01 | 297 |

**HAM-Nat**

| Correlation between HAM-Nat and… | Spearman’s rho | 95% Confidence Interval | n |
| --- | --- | --- | --- |
| MMI | ρ = -.08 | -.21/.07 | 196 |
| Trait EI (TEIQue-SF) | ρ = -.01 | -.15/.11 | 247 |
| Emotion management (STEM) | ρ = -.09 | -.22/.04 | 247 |
| GP psychosocial skills | ρ = -.15 | -.30/.03 | 119 |
| GP suitability | ρ = -.16 | -.33/.02 | 118 |
| OSCE | ρ = -.08 | -.22/.07 | 186 |

* p < .05, ** p < .01

EI = emotional intelligence, GP = general practitioner, GPA = grade point average, HAM-Nat = Hamburg natural sciences test, MMI = multiple mini-interview, OSCE = objective structured clinical examination, STEM = Situational Test of Emotion Management, TEIQue-SF = Trait Emotional Intelligence Questionnaire Short Form
